# Trends in outcomes of 862 giant hiatus hernia repairs over 30 years

**DOI:** 10.1007/s10029-023-02873-1

**Published:** 2023-08-31

**Authors:** C. L. Nguyen, D. Tovmassian, A. Isaacs, S. Gooley, G. L. Falk

**Affiliations:** 1 Department of Upper Gastrointestinal Surgery, Concord Repatriation General Hospital, Concord, NSW 2139 Australia; 2 Department of Surgery, The University of Sydney, Camperdown, NSW 2050 Australia; 3 Sydney Heartburn Clinic, Lindfield, NSW 2070 Australia

**Keywords:** Giant hiatus hernia, Paraesophageal hernia, Repair, Recurrence

## Abstract

**Purpose:**

Laparoscopic giant hiatus hernia repair is technically difficult with ongoing debate regarding the most effective surgical technique. Repair of small hernia has been well described but data for giant hernia is variable. This study evaluated trends in outcomes of laparoscopic non-mesh repair of giant paraesophageal hernia (PEH) over 30 years.

**Methods:**

Retrospective analysis of a single-surgeon prospective database. Laparoscopic non-mesh repairs for giant PEH between 1991 and 2021 included. Three-hundred-sixty-degree fundoplication was performed routinely, evolving into “composite repair” (esophagopexy and cardiopexy to the right crus). Cases were chronologically divided into tertiles based on operation date (Group 1, 1991–2002; Group 2, 2003–2012; Group 3, 2012–2021) with trends in casemix, operative factors and outcomes evaluated. Hernia recurrence was plotted using weighted moving average and cumulative sum (CUSUM) analysis.

**Results:**

862 giant PEH repairs met selection criteria. There was an increasing proportion of “composite repair” after the first decade (Group 1, 2.7%; Group 2, 81.9%; Group 3, 100%; p < 0.001). There were less anatomical hernia recurrence (Group 1, 36.6%; Group 2, 22.9%; Group 3, 22.7%; p < 0.001) and symptomatic recurrence (Group 1, 34.2%; Group 2, 21.9%; Group 3, 7%; p < 0.001) over time. The incidence of anatomical recurrence declined over time, decreasing from 30.8% and plateauing below 17.6% near the study’s end. Median followup (months) in the first decade was higher but followup between the latter two decades comparable (Group 1, 49 [IQR 20, 81]; Group 2, 30 [IQR 15, 65]; Group 3, 24 [14, 56]; p < 0.001). There were 10 (1.2%) Clavien–Dindo grade ≥ III complications including two perioperative deaths (0.2%).

**Conclusion:**

Hernia recurrence rates decreased with increasing case volume. This coincided with the increasing adoption of “composite repair”, supporting the possible improvement in recurrence rates with this approach.

**Supplementary Information:**

The online version contains supplementary material available at 10.1007/s10029-023-02873-1.

## Introduction

The laparoscopic approach to hiatal hernia repair has become widely used since the first laparoscopic fundoplication was described by Dallemagne in 1991 [1]. Hiatus hernia encompass type I (sliding hiatus hernia) and type II–IV (paraesophageal hernias, PEH). A giant hiatus hernia is defined as greater than 30% of stomach herniating through the diaphragmatic hiatus. Giant hiatus hernias are commonly type III or IV, and symptomatic cases generally require surgery. While repair of small symptomatic hernia has been well described with good outcomes, data for giant hernia is more variable [2–4].

Laparoscopic giant PEH repair is a technically difficult operation with significant hernia recurrence rates. A paucity of high-quality evidence has resulted in lack of clarity in guidelines with ongoing debate regarding the most effective surgical technique for repair [5]. Attempts to reduce hernia recurrence rates led to modifications of operative technique including use of mesh repairs and Collis gastroplasty [6, 7]. Larger numbers of giant PEH repairs have been occurring in an aging population with improvement in life expectancy due to better management of comorbidities over time [8]. There is a potential for increased morbidity and mortality amidst the variability in surgical technique [9].

This study contains a large prospective series of giant PEH repair which spans the last three decades. Defining trends in outcomes of a surgical technique over time, in addition to evaluating its safety and efficacy, is helpful to determine adoption of differing techniques into clinical practice. The aim was to evaluate changes in patient outcomes with increasing case volume and evolution of the laparoscopic non-mesh repair technique for giant PEH.

## Methods

### Study design and participants

Data was extracted from a prospectively maintained database of giant hiatus hernia repairs and retrospectively analyzed, with approval from institutional ethics review board (CH62/6/2011-092, LNR/12/CRGH/248, CH62/6/2012-189, LNR/12/CRGH/246) including waiver of consent due to the negligible risk. Patients who underwent primary laparoscopic non-mesh giant PEH repair, performed between March 1991 and November 2021, were included. The operations were predominantly performed by the senior author (GLF) or under direct supervision. Elective and semi-elective cases were included. Indications for surgery included symptomatic PEHs, including acute obstructive symptoms or volvulus, significant gastroesophageal symptoms or dyspnoea. Patients were referred from all over the state of New South Wales, with travel distances between 50 and 700 km.

Inclusion criteria for this study were repair of giant PEH, defined as ≥ 30% of herniated stomach through the diaphragmatic hiatus based on intraoperative findings, and type III or IV hernias. Exclusion criteria were open hernia repairs, use of mesh during a short locum period, Collis gastroplasty used for a short period during an unpublished randomized study, revisional operation and < 12 months of followup (Appendix 1).

**Fig. 1:**
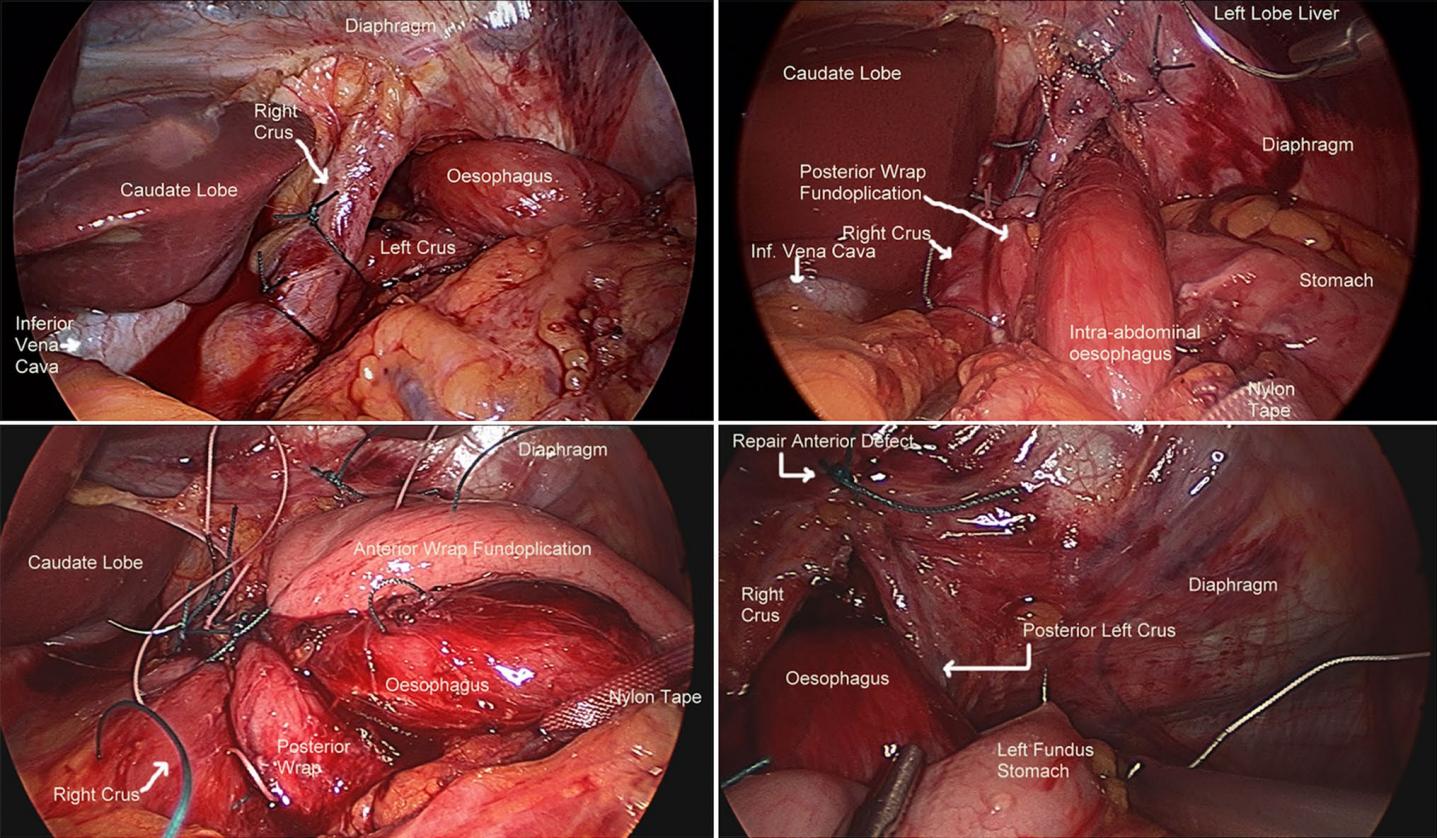
Laparoscopic image of “composite repair” of a giant paraesophageal hernia. Top left image: sutured right and left crura; top right image: posterior fundoplication wrap and cardiopexy; bottom left image: completed fundoplication wrap; bottom right image: sutures to attach left fundus to posterior left crus

The cohort was divided into three groups (tertiles) chronologically based on operation date over the 30-year study period (Group 1, 1991–2002; Group 2, 2003–2012; Group 3, 2012–2021). The technique of hiatus hernia repair involved fundoplication, which was performed routinely and with its nature evolving. Generally a 360-degree fundoplication, similar to what DeMeester [10] and Rossetti and Hell [11] have described, was performed (“Nissen-Rossetti repair”). The significant change over the whole study period was a policy change which occurred in 2007 when a “composite repair”, which incorporated an esophagopexy and cardiopexy to the base of the right crus, was utilised predominantly (Fig. 1) [12].

### Surgical technique

Patients were strongly advised to lose weight preoperatively to body mass index (BMI) < 30. All patients underwent laparoscopic repair of giant PEH without mesh, and with cardio-esophageal junction fixation, either within the fundoplication initially and later by cardiopexy. This involved complete removal of hernia sac from the mediastinum and crural attachments. The esophagus was mobilised in the mediastinum with the aim of establishing a sufficient length to allow tension-free positioning of the gastro-esophageal junction to a length of 2 cm in the abdomen. The crural pillars were sutured deeply, including the central tendinous core, posteriorly and selectively anteriorly. Calibration of the hiatus and fundoplication was performed over a 52 Fr bougie in female and a 56Fr bougie in male patients.

The anaesthetic technique was totally intravenous to avoid postoperative nausea and retching. This was considered paramount having observed multiple immediate recurrences in anti-reflux surgery secondary to retching. Non-steroidal analgesia was preferred postoperatively.

### Outcome variables

The primary outcome was hernia recurrence based on symptoms and endoscopy or radiological imaging. Patient demographics recorded included: age, gender, BMI, ASA (American Society of Anesthesiologists) grade, preoperative symptoms, and hernia type. Intraoperative variables included factors relevant to hiatal closure: hernia size, hiatus size, number of sutures (anterior, posterior and total sutures) in the hiatal repair, and whether there was perceived tension on the hiatus during closure.

Hernia size was estimated by intraoperative assessment and expressed by the percentage of stomach in the mediastinum. Landmarks used at laparoscopy were the pylorus (100% herniation), “crow’s foot” of the incisura (75%) and a point halfway between the crow’s foot and angle of His (50%) (Appendix 2). Hiatus size was classified by surgical estimation as “moderately large”, “large” or “very large”. Tension was assessed as pressure required for apposition of crural pillars by the senior author (GLF), based on significant operative experience.

Postoperative variables included complications graded according to Clavien–Dindo Classification, results of upper endoscopy or barium swallow to detect anatomical recurrence, symptom recurrence, and reinterventions or reoperations. Anatomical recurrence was defined as evidence of any supra-diaphragmatic stomach of either < 2 cm or > 2 cm of intrathoracic stomach, measured vertically from the hiatus.

### Followup

Anatomical followup review with upper endoscopy took place at least once in the first year after surgery and was then planned every 2–3 years subsequently. Patients undertook a barium meal where endoscopy was impractical. Self-administered quality of life (QOL) questionnaires were completed prior to surgery, and within 12 months postoperative. The Gastrointestinal Quality of Life Index (GIQOLI) instrument assesses gastrointestinal-specific health-related QOL, with higher scores (range 0–144) suggesting better gastrointestinal health-related QOL [13].

### Statistical analysis

Fisher’s exact test was used to evaluate differences in tertiles in relation to casemix characteristics, complications, and hernia recurrence. Analysis of variance (ANOVA) was used to determine any statistically significant differences between the followup time of patients in the different tertiles. Tukey multiple pairwise-comparisons were performed to determine if the difference between specific pairs of groups were statistically significant. Two-tailed Student’s t test was used to compare continuous variables. Sensitivity analyses were performed to confirm that observed effects were not a product of grouping decisions, including an adjustment for different group sizes. Continuous variables were presented as means with standard deviation (SD) or as median with interquartile ranges (IQR), and dichotomous and categorical data as frequencies with percentages. A p value < 0.05 was considered significant. The proportion of patients with anatomical hernia recurrence was estimated using Kaplan–Meier curves. Statistical analysis was performed with RStudio, version 2022.07.2.

The incidence of hernia recurrence was calculated for each individual case using centered linear weighted moving average analysis, with a window size of 51 cases. A window size of 51 meant that 25 cases on each side of the case for which the incidence is calculated for was used. This was so outcomes of cases operated early in the series did not influence the incidence of cases operated later in the study period. Cumulative sum (CUSUM) analysis was used to plot the incidence of hernia recurrence over the number of caseload. CUSUM is a sequential analysis technique which detects deviation of individual values or subgroup mean from the adjusted target value in the data series. It has been used as a graphical representation of the trend in outcomes of a series of consecutive procedures performed over time, and designed to detect change in performance associated with a potential unacceptable rate of adverse outcome [14].

## Results

### Casemix characteristics

**Fig. 2:**
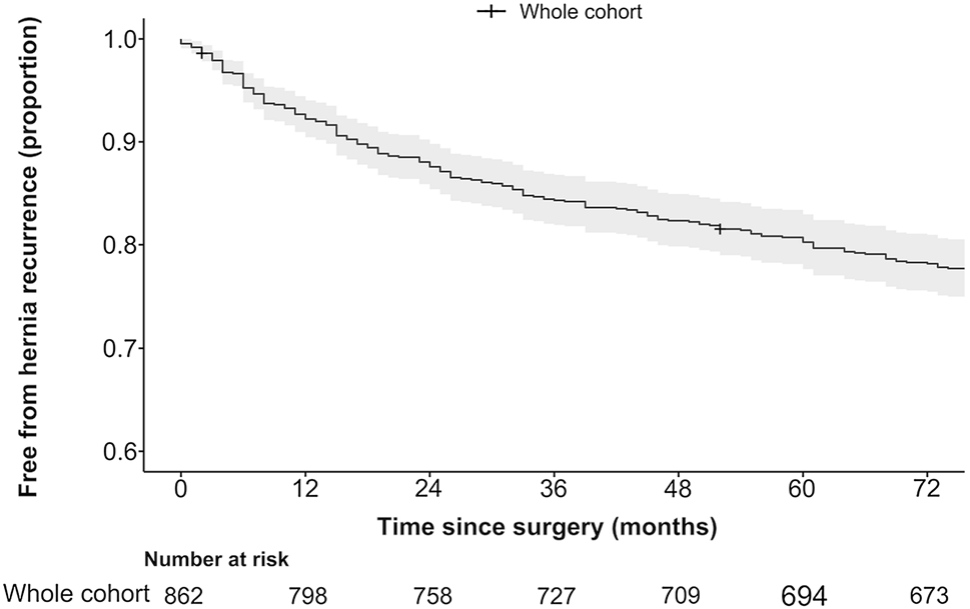
Kaplan–Meier curve for freedom from anatomical hernia recurrence over 6 years for the whole cohort. The solid line is the point estimate of the proportion of patients free from hernia recurrence. The grey outline is the 95% confidence interval

A total of 962 giant PEH repairs were performed over the 30-year study period. Following selection criteria, 862 laparoscopic primary non-mesh repairs of giant PEH were analyzed. The mean age was 69.3 years (SD 10.6). The majority (65.8%) were female with a mean BMI of 27.9 (SD 4.3) and median ASA of 3 (IQR 2–3). The most common symptoms were dyspnoea (65.9%), chest or epigastric pain (52.6%), heartburn (52.6%), regurgitation (50.5%) and dysphagia (50.5%). Most were type III (87.4%) with median hernia size of 66% (IQR 45, 80), and mostly with “large” hiatus size (76%) (Appendix 3). Median followup was 33 months (IQR 16, 68). Overall rate of anatomical hernia recurrence was 27.4%. Median time to anatomical hernia recurrence was 26 months (IQR 10, 59) (Fig. 2). Overall rate of revision surgery for hernia recurrence was 3.8%.

## Analysis of patient and operative characteristics in tertiles

Casemix variables by groups chronologically divided into tertiles are presented in Table 1. More patients had preoperative dyspnoea and cough over time. There were more patients with female gender (Group 1, 61.3%; Group 2, 64.9%; Group 3, 71.1%; p = 0.04) and ASA > 3 (Group 1, 61.7%; Group 2, 50.4%; Group 3, 67.6%; p < 0.001) operated on in the most recent decade. A greater proportion of cases had “very large” hiatus size over the three decades (Group 1, 5.2%; Group 2, 10.4%; Group 3, 38.3%; p < 0.001).

**Table 1:** Casemix variables by groups chronologically divided into tertiles

	Group 1, N = 287	Group 2, N = 288	Group 3, N = 287	p value^c^
Patient characteristics				
Age, years, N (%)				0.24
≤ 70	144 (50.2%)	144 (50%)	129 (44.9%)	
> 70	143 (49.8%)	144 (50%)	158 (55.1%)	
Gender, N (%)				**0.04**
Male	111 (38.7%)	101 (35.1%)	83 (28.9%)	
Female	176 (61.3%)	187 (64.9%)	204 (71.1%)	
BMI, N (%)				0.35
≤ 30	139 (48.4%)	135 (46.9%)	169 (58.9%)	
> 30	80 (27.9%)	81 (28.1%)	78 (27.2%)	
ASA, N (%)				**< 0.001**
≤ 3	110 (38.3%)	136 (47.2%)	88 (30.7%)	
> 3	177 (61.7%)	145 (50.4%)	194 (67.6%)	
Symptoms present, N (%)				
Dyspnoea	170 (59.2%)	198 (68.8%)	200 (69.7%)	** < 0.001**
Chest/epigastric pain	142 (49.9%)	149 (51.7%)	162 (56.5%)	0.34
Heartburn	152 (53%)	139 (48.3%)	162 (56.5%)	0.22
Regurgitation	151 (52.6%)	129 (44.8%)	155 (54%)	0.14
Dysphagia	146 (50.9%)	129 (44.8%)	160 (55.8%)	0.19
Cough	50 (17.4%)	74 (25.7%)	107 (37.3%)	**< 0.001**
Hernia characteristics				
Hernia type IV, N (%)				0.13
Absent	278 (96.9%)	269 (93.4%)	275 (95.8%)	
Present	9 (3.1%)	19 (6.6%)	12 (4.2%)	
Hiatus size “very large”, N (%)				**< 0.001**
Absent	272 (94.8%)	258 (89.6%)	177 (61.7%)	
Present	15 (5.2%)	30 (10.4%)	110 (38.3%)	
Hernia size^a^, N (%)				0.11
≤ 75%	156 (54.4%)	191 (66.3%)	169 (58.9%)	
> 75%	131 (45.6%)	97 (33.7%)	118 (41.1%)	
Operative characteristics				
Composite repair^b^				**< 0.001**
Absent	279 (97.2%)	52 (18.1%)	0	
Present	8 (2.8%)	236 (81.9%)	287 (100%)	
Hiatus closure under tension, N (%)				**< 0.001**
Absent	275 (95.8%)	249 (86.5%)	255 (88.9%)	
Present	12 (4.2%)	39 (13.5%)	32 (11.1%)	
Total crural sutures, N (%)				**< 0.001**
≤ 3	22 (7.67%)	3 (1.04%)	22 (7.67%)	
> 3	234 (81.5%)	275 (95.5%)	264 (92%)	
Anterior crural sutures, N (%)				**0.01**
≤ 3	250 (87.1%)	260 (90.3%)	272 (94.8%)	
> 3	3 (1.1%)	18 (6.3%)	14 (4.9%)	
Posterior crural sutures, N (%)				**< 0.001**
≤ 3	111 (38.7%)	215 (74.7%)	225 (78.4%)	
> 3	142 (49.5%)	63 (21.9%)	61 (21.3%)	

*BMI* body mass index, *ASA* American Society of Anesthesiologists

^a^Percentage in mediastinum

^b^Incorporated 360° fundoplication with oesophagopexy and cardiopexy to right crus

^c^Fisher’s exact test

Bold values represents the statistically significant i.e < 0.05

There was a significant increase in proportion of “composite repair” after the first decade (Group 1, 2.7%; Group 2, 81.9%; Group 3, 100%; p < 0.001). A greater proportion of cases had a hiatus closure under tension in the latter two decades (Group 1; 4.2%; Group 2, 13.5%; Group 3, 11.1%; p < 0.001). The latter two decades also saw a greater proportion of cases using total crural sutures > 3 (Group 1, 81.5%; Group 2, 95.5%; Group 3, 92%; p < 0.001) with more cases using > 3 anterior crural sutures (Group1, 1.1%; Group 2, 6.3%; Group 3, 4.9%; p = 0.01) and ≤ 3 posterior crural sutures (Group 1, 38.7%; Group 2, 74.7%; Group 3, 78.4%; p < 0.001) compared with the first decade.

## Analysis of patient outcomes in tertiles

Outcomes of patients by groups chronologically divided into tertiles are presented in Table 2. There were less anatomical hernia recurrence (Group 1, 36.6%; Group 2, 22.9%; Group 3, 22.7%; p < 0.001) and symptomatic recurrence (Group 1, 34.2%; Group 2, 21.9%; Group 3, 7%; p < 0.001) over the three decades of operating.

**Table 2:** Outcomes of patients by groups chronologically divided into tertiles

	Group 1, N = 287	Group 2, N = 288	Group 3, N = 287	p value^c^
Anatomical recurrence, N (%)				**< 0.001**
Present	105 (36.6%)	66 (22.9%)	65 (22.6%)	
Absent	182 (63.4%)	222 (77.1%)	222 (77.4%)	
Hernia recurrence size, N (%)				0.21
> 2 cm	37 (35.2%)	32 (48.5%)	28 (43.1%)	
< 2 cm	68 (64.8%)	34 (51.5%)	37 (56.9%)	
Symptom recurrence, N (%)				**< 0.001**
Present	98 (34.1%)	63 (21.9%)	20 (7%)	
Absent	189 (65.9%)	225 (78.1%)	267 (93%)	
Revision, N (%)				**0.01**
Present	3 (1.1%)	14 (4.9%)	16 (5.6%)	
Absent	284 (98.9%)	274 (95.1%)	271 (94.4%)	
Complication^a^, N (%)				0.17
Present	4 (1.4%)	1 (0.35%)	5 (1.7%)	
Absent	283 (98.6%)	287 (99.7%)	282 (98.3%)	
Followup, median months (IQR)	49 (20, 81)	30 (15, 65)	24 (14, 56)	**< 0.001**
QOL GIQLI score^b^, mean (SD)				
Preoperative	76.1 (36.6)	96.2 (20.7)	74.5 (39.7)	0.29^d^
At 12 months postoperative	108 (20.1)	110.4 (21.8)	108.8 (22.1)	0.44^d^

*SD* standard deviation, *IQR* interquartile range

^a^Clavien–Dindo grade III and above

^b^*GIQLI* Gastrointestinal Quality of Life Index score (Max score of 144)

^c^Fisher’s exact test; ^d^ANOVA

Bold values represents the statistically significant i.e < 0.05

**Fig. 3:**
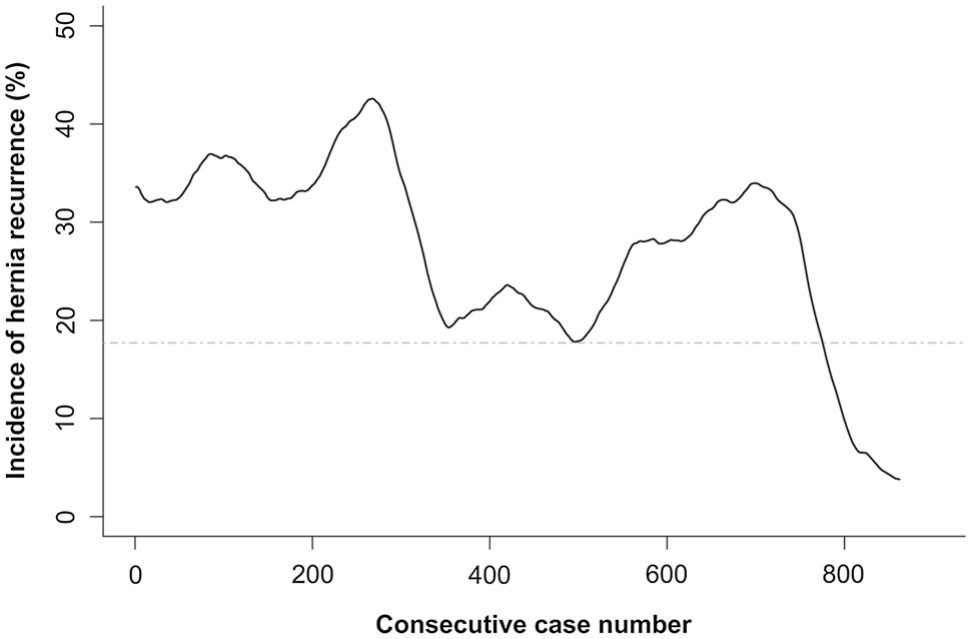
Incidence of anatomical hernia recurrence in laparoscopic non-mesh repair of giant paraesophageal hernia. The solid line is the observed incidence of anatomical hernia recurrence. The horizontal dashed line is the plateau anatomical hernia recurrence incidence of 18%

The incidence of anatomical hernia recurrence declined over time from 30.8% and plateauing below 17.6% near the end of the study period. There were two peaks in incidence of hernia recurrence of 44.3% and 34.6% during the first and third tertiles, respectively (Fig. 3).

There were significantly more revision operations over time (Group 1, 1.1%; Group 2, 4.9%; Group 3, 5.6%; p < 0.001). The symptoms prompting reoperation included dysphagia, reflux and pain, with a demonstratable hernia recurrence on upper endoscopy or radiological imaging.

There was no significant difference in the number of postoperative complications across the three decades. Significant complications (Clavien–Dindo grade ≥ III) occurred in 10 patients (1.2%), including two perioperative deaths (0.2%). There was one postoperative death on day four due to small bowel ischemia on a background of pre-existing atrial fibrillation. The other postoperative death was from a myocardial infarction while in intensive care. One patient had an infected mediastinal haematoma managed with drainage and antibiotics but subsequently died many months following surgery. One patient required conversion to open surgery for haemostasis of a bleeding branch from the left gastric artery. There was an immediate hernia recurrence requiring reoperation, an esophageal injury repaired by thoracotomy, an intrabdominal sepsis from a subphrenic abscess requiring return to theatre for laparotomy, and a case of respiratory failure requiring reintubation.

Median followup for patients in the first decade was significantly higher than those in the latter two decades (Group 1, 49 months [IQR 20, 81]; Group 2, 30 [IQR 15, 65]; Group 3, 24 [14, 56]). Pairwise-comparisons did not demonstrate a significant difference between groups 2 and 3.

Preoperative mean QOL scores using GIQLI were reduced in patients with giant PEH when compared to a perfect score. Significant improvement in these scores was reported at 12 months postoperative for each tertile. Survey participation rate was 80.5% (694/862).

## Subgroup analysis of “composite repair”

There were 531 (61.6%) patients that underwent “composite repair” compared to 331 (38.4%) that underwent surgery without. The patient demographics and hernia characteristics were mostly similar between the two groups except the “composite repair” group had higher proportion of females, preoperative dyspnoea and cough, hiatus size of “very large”, and hiatus closure under tension on subgroup analysis (Appendix 4). Median followup for patients that underwent “composite repair” was less compared to those that did not (24 months [IQR 15, 38.5] versus 45 months [IQR 20, 71], respectively, p < 0.001). Patients that underwent “composite repair” had less anatomical hernia recurrence (22.2% versus 35.6%, p < 0.001, respectively) and symptomatic recurrence (15.1% versus 30.5%, p < 0.001, respectively) compared to those that did not have “composite repair” (Table 3).

**Table 3:** Outcomes of “composite repair” and “non-composite repair” cohorts

	Composite repair, N = 531	Non-composite repair, N = 331	p value^c^
Anatomical recurrence, N (%)			** < 0.001**
Present	118 (22.2%)	118 (35.6%)	
Absent	413 (77.8%)	213 (64.4%)	
Hernia recurrence size, N (%)			1
> 2 cm	49 (41.5%)	48 (40.7%)	
< 2 cm	69 (58.5%)	70 (59.3%)	
Symptom recurrence, N (%)			** < 0.001**
Present	80 (15.1%)	101 (30.5%)	
Absent	451 (84.9%)	230 (69.5%)	
Revision, N (%)			0.43
Present	23 (4.3%)	10 (3%)	
Absent	508 (95.7%)	321 (97%)	
Complication^a^, N (%)			1
Present	6 (1.1%)	4 (1.2%)	
Absent	525 (98.9%)	327 (98.8%)	
QOL GIQLI score^b^, mean (SD)			
Preoperative	83.3 (34.2)	75.1 (43.7)	0.38^d^
At 12 months postoperative	110.2 (20.6)	105.7 (25.3)	0.41^d^

*SD* standard deviation

^a^Clavien–Dindo grade III and above

^b^GIQLI, Gastrointestinal Quality of Life Index score (Max score of 144)

^c^Fisher’s exact test

^d^Two-tailed t test

Bold values represents the statistically significant i.e < 0.05

## Discussion

Technical aspects of laparoscopic repair of giant PEH have been at the discretion of individual surgeons with wide variations in techniques including hiatal repair with and without mesh as demonstrated in a survey of European surgeons and large series previously published (Table 4) [15–22]. Giant PEH can cause debilitating symptoms and result in significant complications with mortality rates up to 16% over 4 years [2]. Larger numbers of giant PEH repairs are occurring in an aging population with the improvement in life expectancy achieved from advances in medicine [9]. This makes adopting an effective surgical technique important.

**Table 4:** Hernia recurrence rates in large series (> 100) of giant paraesophageal hernia repair

Study, year	Patients, N	Study type	Repair type	Median followup, months	Anatomical recurrence, N (%)	Recurrence size < 2 cm/> 2 cm, N (%)	Symptomatic recurrence, N (%)	Complications^b^, N (%)	Revision, N (%)
Luketich [21], 2010	662	Retrospective	Lap, mesh, non-mesh	30 (IQR 17, 56)	70/445 (15.7%)	Not reported	Not reported	Not reported	21 (3.2)
Chang [20], 2016	221	Retrospective	Lap, mesh	14.5 (mean, SD 11)	8 (3.6%)	6 (75%)/ 2 (25%)	Not reported	4 (1.8%)	1 (0.5)
Antiporda [2], 2017	202	Prospective	Lap, mesh	7 (range 3–72)	69 (34.2%)	Not reported	20 (9.9%)	3 (1.5%)	10 (5%)
Stringham [23], 2017	106	Prospective	Lap, open, non-mesh	12 (IQR, 13, 15)	33 (32.7%)	14 (42.4%)/ 19 (57.5%)	37 (36.6%)	8.5%	4 (3.8%)
El Lakis [3], 2017	524	Prospective	Lap, open, non-mesh	4.3	54/313 (17.3%)	54 (100%)/ 0	Not reported	11 (2.1%)	4 (0.8%)
Quinn [22], 2019	178	Prospective	Mixed^a^	35 (range 12–238)	Not reported	Not reported	15 (8.4%)	1.6% mortality	13 (7.3%)
Current study, 2023	862	Retrospective	Lap, non-mesh	33 (IQR 16, 68)	236 (27.4%)	139 (58.9%)/ 97 (41.1%)	181 (21%)	10 (1.2%)	33 (3.8%)

*Lap* laparoscopic, *SD* standard deviation, *IQR* interquartile range

^a^Mix of open, laparoscopic, mesh and non-mesh repairs

^b^Clavien–Dindo grade III and above

This current study evaluated changes in patient outcomes with laparoscopic non-mesh repair of giant PEH over 862 cases across three decades. Hernia recurrence rates decreased with increasing case volume possibly due to the transition towards a “composite repair”, which involved 360-degree fundoplication with esophagopexy and cardiopexy to the right crural pillar. The median followup for those that had “composite repair”, however, was significantly less than those with “Nissen-Rossetti repair”. This was partly due to a much greater proportion of “composite repair” occurring in the most recent decades which shortens potential followup.

The demographics of this patient cohort are similar to other studies, with a female predominance and elderly population [3, 18]. There was a greater proportion of patients with preoperative dyspnoea and cough over time. The apparent change in rate may represent increasing awareness of dyspnoea as a symptom of PEH, however it could also be due to the increasing size of hernias over time. Significant rates of dyspnoea (77.1%) and pulmonary aspiration (61.4%), based on symptoms and reflux micro-aspiration scintigraphy, have previously been shown to occur in the presence of giant PEH [23]. Respiratory symptoms can be a presenting symptom of large hernia due to its direct mechanical effect on the lung and pulmonary micro-aspirations as well as cardiac inflow obstruction which improves following hernia repair [24].

There were more patients with significant comorbidities operated on during the most recent decade which likely reflects the increasing ageing population [9]. Many patients presenting with giant PEH are elderly, and it has been recognized that laparoscopic repair is safe and effective, comparable with younger patients, when appropriately selected [8, 25]. There was also an increasing number of patients with “very large” hiatus size over the three decades. The rate of complications and hernia recurrence remained low over the study period despite these trends, which would have been expected to increase recurrence rates.

Revision surgery was offered to patients with hernia recurrence and severe persistent symptoms despite medical treatment. The rate of reoperation was significantly greater in the latter two decades. It could, however, be due to the increased life expectancy and improved QOL of patients which allowed major revision surgery that would once have not been considered to be safe. It is possible that, in the early group, revisions were not as readily offered to elderly comorbid patients. Revision surgery rates overall (3.8%) were low, which is similar to previous studies [18].

Outcomes with the surgical technique demonstrated fair symptom control, and recurrence and reoperation rates comparable to other techniques. None of the various techniques of giant PEH repair have yet to be shown to be superior [19, 26–29]. Recurrence rates reported in the literature vary greatly (8–66%). This is partly due to use of varying definitions of recurrence, with some studies only reporting hiatus hernias over 2 cm of intrathoracic stomach measured vertically from hiatus [19, 30]. The rate of anatomical recurrence in this current study (27.4%) is likely to reflect true rates as patients were followed up endoscopically or radiologically, and all recurrences were documented regardless of size.

In this study, the incidence of anatomical hernia recurrence declined over time. There were two peaks in incidence of hernia recurrence, one during the first decade and the second during the third decade. The first peak could not be entirely explained by selection of patients. It could have been due to the modification in technique to incorporate a “composite repair”. The second peak could be attributable to the higher proportion of cases with “very large” hiatus size, as it is unlikely to be explained by any learning curve related to the “composite repair” technique as it occurred near the end of the study period.

The median time to recurrence diagnosis was 26 months, which is within the range reported for suture, and synthetic or biologic mesh repairs [18, 31]. Repair of the hiatus was done in this study with placement of two to four posterior sutures of non-absorbable material and anterior hiatus sutures in the central tendon of the diaphragm. This anterior repair enabled recruitment of the left hemi-diaphragm for less tension on hiatus closure by flattening the diaphragmatic dome, as the left crus moved towards the more fixed right crus. Deep suturing of the right crural pillar avoided tearing of this structure. Mesh repair was not utilised in this series.

Use of reinforced mesh crural repair is still controversial regarding prevention of hernia recurrence and risk of mesh-related complications [12]. Mesh repair has not been definitively found to offer significant advantage over sutured hiatal closure. Recent meta-analyses have demonstrated that both techniques deliver good and comparable clinical outcomes [19, 26–29]. No significant advantages were reported in a recent randomized controlled trial (RCT) for mesh repair at up to 5 years followup [32]. Any potential advantages of mesh repair should be balanced with the risk of complications, and potential difficulties at reoperation [33].

“Short esophagus” used to be a common manifestation of peptic stricture in gastroesophageal reflux disease, and Collis gastroplasty was historically an important part of anti-reflux surgery and hiatus hernia repair [34]. Widespread use of proton-pump inhibitors has nearly eradicated peptic strictures. Collis gastroplasty however remains utilised in some units with reported prevalence up to 66% [18, 22, 35]. Apart from several early cases that underwent Collis gastroplasty in this series, none required it since then, which is a similar trend to other reports [18, 36].

With adequate esophageal mobilization there is an area of dense fascia in the mid-mediastinum, which once divided allows the esophagus to be retracted intra-abdominally more readily. Additionally, dissection of the posterior vagus nerve from the esophagus and posterior region of the stomach’s bare area, often allows further reduction of the cardio-esophageal junction intra-abdominally [12]. The concept of short esophagus may be inconsequential given that recurrence rates in this study were fairly low and similar to other published series [18, 36].

For the fixation and fundoplication, the superior aspect of the posterior stomach was passed around the esophagus, creating a soft wrap ensuring no tension on the stomach or esophagus. Two sutures incorporating cardio-esophageal junction, posterior fundus, median arcuate ligament and repaired crus were placed. Total fundoplication was then completed by three sutures through the left-sided anterior fundus, esophagus and right-sided posterior fundus. Further sutures were placed attaching the left fundus to the posterior left crus. The presence of a massive defect in the anti-reflux mechanism of the hiatus, associated with PEH as demonstrated in RCT data, is persuasive of the desirability of an anti-reflux procedure [37].

Hernia recurrence and crural defects have been previously demonstrated to increase variably over time. Anterior crural defects in particular increase over time, which suggests deficiency of the central tendon being the primary mechanism [38]. There was a trend in this current study towards use of a greater number of total crural sutures, including more anterior sutures. A robust anterior repair of the central tendon in the larger hiatus hernia could possibly help reduce the risk of late recurrence.

“Telescoping” of the cardio-esophageal junction through an intact hiatus and fundoplication has been shown to be an early event in hernia recurrence [38]. This could possibly be reduced with a “composite repair”. This approach, involving fixation of cardio-esophageal junction to the right crus along with the fundoplication, was by far the most significant change in operative variable pertinent to repair observed during the study period. There was a significant and consistent trend towards use of this technique after the first decade of operating. Subgroup analysis demonstrated that cases utilising this approach had better outcomes in terms of both anatomical and symptomatic hernia recurrence, although median review was shorter.

Repair of giant PEH remains a difficult operation on a number of bases. It is difficult technically as well as often being performed in an elderly comorbid population with significant surgical risk and recurrence rates. Recurrence detected objectively using endoscopy or radiological imaging remains significant, even if a correlation with symptoms is not commonly present. Attempts to improve closure of the hiatal defect, through use of mesh, may come at the cost of increased risk of mesh-related complications [32]. This study found that a non-mesh repair provided fair symptom relief. The majority of hernia recurrence were small and asymptomatic. Postoperative mortality and major morbidity were low.

Strengths of this study include the large number of operations performed in a high-volume setting with endoscopic followup and QOL surveys. Limitations include lack of operation duration data which may have been useful as repair of giant herniae are technically complex with significant mediastinal dissection and longer operation duration often required. There was also a statistically significant difference in the length of followup between the three groups. Followup was significantly longer, as expected, in the first group compared to the latter two which is a potential major bias. Followup for the latter two groups, however, was not found to be statistically significant and so some conclusion can be drawn about the decreasing recurrence rates over time, albeit not as strong. Long-term followup for this patient population was very difficult to obtain because of the advanced age of patients undergoing surgery. These numbers are comparable to previous large series containing at least 100 giant PEH repairs and encountered similar issues with followup data due to extremes of age [2, 3, 20–23]. Elderly patients at the time of operation, and especially those with significant comorbidities, operated on during the early periods were more likely to have shorter followup as they passed away. Lack of comparable followup time to review did not allow for accurate comparison of “composite repair” and “Nissen-Rossetti repair” techniques. Symptomatic patients could arguably have been more likely to return for review compared with patients who remained asymptomatic which may falsely degrade results. It was not possible to accurately identify any patients that may have had a revision operation elsewhere which was another potential bias. The results also may be less generalizable as trends in outcomes can more easily be influenced by chance or changing casemix in single-center studies compared to studies with pooled data [14].

Non-mesh repair offered results similar to other techniques. Defining trends in outcomes over time, in addition to a surgical technique’s safety and efficacy, is helpful to determine adoption into clinical practice, and improve outcomes. In the largest personal series to date, it was found that laparoscopic non-mesh repair of giant PEH was associated with good outcomes. Hernia recurrence rates decreased with increasing case volume. This coincided with the increasing adoption of “composite repair”, supporting the possible improvement in recurrence rates with this approach.

### Supplementary Information

Below is the link to the electronic supplementary material.

Supplementary file1 (DOCX 101 KB)

Supplementary file2 (TIFF 554 KB)

## Data Availability

A statement about data availability on request from the corresponding author.
