# Supplementary material for: Trends in outcomes of 862 giant hiatus hernia repairs over 30 years
Source: Hernia. 2023 Aug 31;27(6):1543–53. doi: 10.1007/s10029-023-02873-1 (PMC10700453; doi:10.1007/s10029-023-02873-1)
Supplement: Supplementary file 1 — Supplementary file1 (DOCX 101 KB) [file 10029_2023_2873_MOESM1_ESM.docx]

**SUPPLEMENTARY MATERIAL**

Appendix 1. Selection criteria for laparoscopic non-mesh repair of giant paraesophageal hernia study


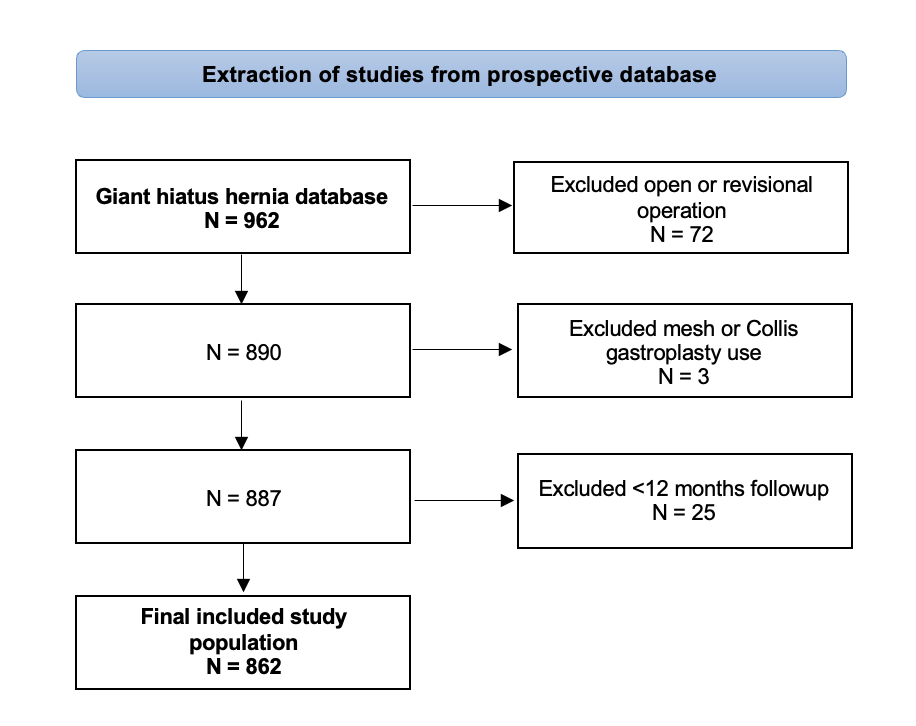


Appendix 2. Hernia size estimation by intraoperative assessment expressed by percentage of stomach in mediastinum. Stomach depicted below the diaphragm. Line B depicts 75% and line A depicts 50%.

|  | **Whole cohort,**  **N = 862** |
| --- | --- |
| **Patient characteristics** |  |
| Age, mean (SD) | 69.3 (10.6) |
| Gender, N (%) |  |
| Female | 567 (65.8%) |
| Male | 295 (34.2%) |
| BMI, mean (SD) | 27.9 (4.3) |
| ASA, median (IQR) | 3 (2-3) |
| Barrett’s esophagus, N (%) | 174 (20.2%) |
| Symptoms, N (%) |  |
| Dyspnoea | 568 (65.9%) |
| Chest/ epigastric pain | 453 (52.6%) |
| Heartburn | 453 (52.6%) |
| Regurgitation | 435 (50.5%) |
| Dysphagia | 435 (50.5%) |
| Cough | 231 (26.8%) |
| **Hernia characteristics** |  |
| Hernia type, N (%) |  |
| II | 69 (8%) |
| III | 753 (87.4%) |
| IV | 40 (4.6%) |
| Hernia size^1^, median % (IQR) | 66 (45, 80) |
| Hiatus size, N (%) |  |
| Moderate | 52 (6%) |
| Large | 655 (76%) |
| Very large | 155 (18%) |
| **Operative characteristics** |  |
| “Composite repair”^2^, N (%) |  |
| Present | 531 (61.6%) |
| Absent | 331 (38.4%) |
| Total crural sutures, median (IQR) | 4 (3-4) |
| Anterior crural sutures | 1 (1-2) |
| Posterior crural sutures | 2 (2-3) |
| Hiatus closure under tension, N (%) | 83 (9.6%) |
|  |  |

Appendix 3. Characteristics of patients undergoing laparoscopic repair of giant paraesophageal hernia

^1^Percentage in mediastinum

^2^Incorporated 360° fundoplication with oesophagopexy and cardiopexy to right crus

BMI, Body mass index; ASA, American Society of Anesthesiologists; SD, Standard deviation; IQR, Interquartile range

Appendix 4. Casemix variables by “composite repair” and “non-composite repair” cohorts

|  | **Composite repair,**  **N = 531** | **Non-composite repair,**  **N = 331** | **p value** |
| --- | --- | --- | --- |
| **Patient characteristics** |  |  |  |
| Age, years, N (%) |  |  | .12 |
| ≤70 | 243 (45.8%) | 170 (51.4%) |  |
| >70 | 284 (53.5%) | 158 (47.7%) |  |
| Gender, N (%) |  |  | **.003** |
| Male | 161 (30.3%) | 134 (40.5%) |  |
| Female | 370 (69.7%) | 197 (59.5%) |  |
| BMI, N (%) |  |  | .72 |
| ≤30 | 276 (52%) | 167 (50.5%) |  |
| >30 | 153 (28.8%) | 86 (26%) |  |
| ASA, N (%) |  |  | .8 |
| ≤3 | 207 (39%) | 127 (38.4%) |  |
| >3 | 314 (59.1%) | 202 (61%) |  |
| Symptoms present, N (%) |  |  |  |
| Dyspnoea | 368 (69.3%) | 200 (60.4%) | **<.001** |
| Chest/ epigastric pain | 299 (56.3%) | 154 (46.5%) | **<.001** |
| Heartburn | 282 (53.1%) | 171 (51.7%) | .24 |
| Regurgitation | 273 (51.4%) | 162 (48.9%) | .18 |
| Dysphagia | 278 (52.4%) | 157 (47.4%) | .1 |
| Cough | 169 (31.8%) | 62 (18.7%) | **<.001** |
| **Hernia characteristics** |  |  |  |
| Hernia type IV, N (%) |  |  | .34 |
| Absent | 503 (94.7%) | 319 (96.4%) |  |
| Present | 28 (5.3%) | 12 (3.6%) |  |
| Hiatus size “very large” , N (%) |  |  | **<.001** |
| Absent | 397 (74.8%) | 310 (93.7%) |  |
| Present | 134 (25.2%) | 21 (6.3%) |  |
| Hernia size^1^, N (%) |  |  | .17 |
| ≤75% | 328 (61.8%) | 188 (56.8%) |  |
| >75% | 203 (38.2%) | 143 (43.2%) |  |
| **Operative characteristics** |  |  |  |
| Total crural sutures, N (%) |  |  | .17 |
| ≤3 | 25 (4.7%) | 22 (6.6%) |  |
| >3 | 497 (93.6%) | 276 (83.4%) |  |
| Anterior crural sutures, N (%) |  |  | **.01** |
| ≤3 | 492 (92.7%) | 290 (87.6%) |  |
| >3 | 30 (5.6%) | 5 (1.5%) |  |
| Posterior crural sutures, N (%) |  |  | **<.001** |
| ≤3 | 398 (75%) | 153 (46.2%) |  |
| >3 | 124 (23.4%) | 142 (42.9%) |  |
| Hiatus closure under tension, N (%) |  |  | **.002** |
| Absent | 466 (87.8%) | 313 (94.6%) |  |
| Present | 65 (12.2%) | 18 (5.4%) |  |
|  |  |  |  |

^1^Percentage in mediastinum

BMI, Body mass index; ASA, American Society of Anesthesiologists
