# Supplementary figures and images for: Trends in outcomes of 862 giant hiatus hernia repairs over 30 years
Source: Hernia. 2023 Aug 31;27(6):1543–53. doi: 10.1007/s10029-023-02873-1 (PMC10700453; doi:10.1007/s10029-023-02873-1)

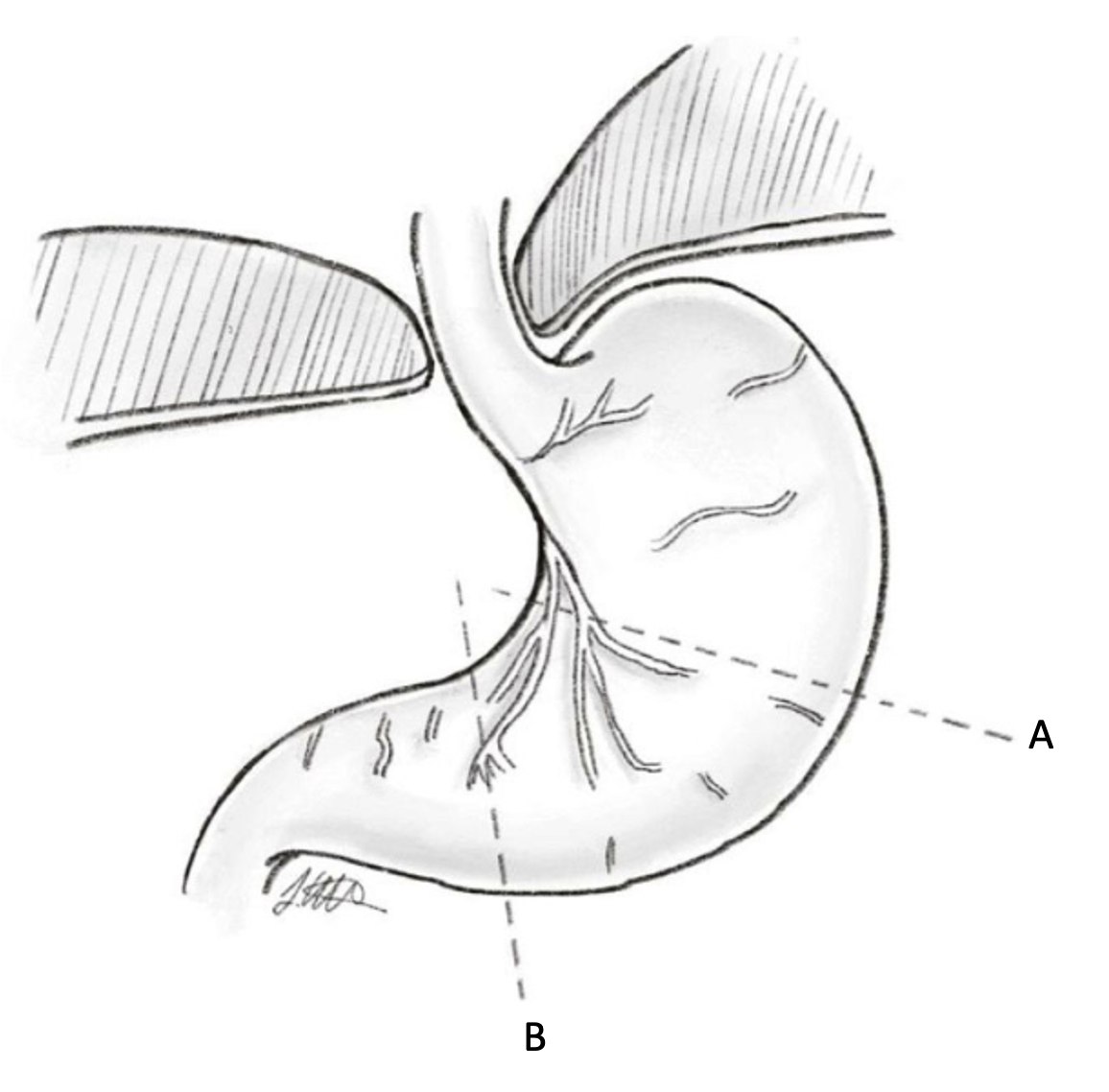

Supplement: Supplementary file 2 — Supplementary file2 (TIFF 554 KB) [file 10029_2023_2873_MOESM2_ESM.tiff]
